# DNA Methylation Data-Based Classification and Identification of Prognostic Signature of Children With Wilms Tumor

**DOI:** 10.3389/fcell.2021.683242

**Published:** 2021-12-24

**Authors:** Fucai Tang, Zeguang Lu, Hanqi Lei, Yongchang Lai, Zechao Lu, Zhibiao Li, Zhicheng Tang, Jiahao Zhang, Zhaohui He

**Affiliations:** ^1^ Department of Urology, The Eighth Affiliated Hospital, Sun Yat-sen University, Shenzhen, China; ^2^ The Second Clinical College of Guangzhou Medical University, Guangzhou, China; ^3^ Department of Urology, The Seventh Affiliated Hospital of Sun Yat-Sen University, Shenzhen, China; ^4^ Department of Urology, Sun Yat-sen Memorial Hospital of Sun Yat-sen University, Guangzhou, China; ^5^ The First Clinical College of Guangzhou Medical University, Guangzhou, China; ^6^ The Third Clinical College of Guangzhou Medical University, Guangzhou, China; ^7^ The Sixth Clinical College of Guangzhou Medical University, Guangzhou, China

**Keywords:** DNA methylation, wilms tumor, target database, children, prognosis

## Abstract

**Background:** As an epigenetic alteration, DNA methylation plays an important role in early Wilms tumorigenesis and is possibly used as marker to improve the diagnosis and classification of tumor heterogeneity.

**Methods:** Methylation data, RNA-sequencing (RNA-seq) data, and corresponding clinical information were downloaded from the Therapeutically Applicable Research to Generate Effective Treatments (TARGET) database. The prognostic values of DNA methylation subtypes in Wilms tumor were identified.

**Results:** Four prognostic subtypes of Wilms tumor patients were identified by consensus cluster analysis performed on 312 independent prognostic CpG sites. Cluster one showed the best prognosis, whereas Cluster two represented the worst prognosis. Unique CpG sites identified in Cluster one that were not identified in other subtypes were assessed to construct a prognostic signature. The prognostic methylation risk score was closely related to prognosis, and the area under the curve (AUC) was 0.802. Furthermore, the risk score based on prognostic signature was identified as an independent prognostic factor for Wilms tumor in univariate and multivariate Cox regression analyses. Finally, the abundance of B cell infiltration was higher in the low-risk group than in the high-risk group, based on the methylation data.

**Conclusion:** Collectively, we divided Wilms tumor cases into four prognostic subtypes, which could efficiently identify high-risk Wilms tumor patients. Prognostic methylation risk scores that were significantly associated with the adverse clinical outcomes were established, and this prognostic signature was able to predict the prognosis of Wilms tumor in children, which may be useful in guiding clinicians in therapeutic decision-making. Further independent studies are needed to validate and advance this hypothesis.

## Introduction

Wilms tumor (WT; nephroblastoma) is the most frequently identified renal tumor in the genitourinary tract of children, accounting for 5% of all childhood malignancies (American Cancer Society, 2020). Depending on the presence or absence of reversionary atrophy, the histological types of WT can be divided into two broad categories: favorable histology (FH) and unfavorable histology (UH) (Perlman, 2005). Despite remarkable achievements in therapeutic strategies, the survival rate for certain patient subgroups remains well below 90%, including those classified as UH (Dome et al., 2015). Nearly 25% of survivors experience severe chronic health conditions after WT treatment (Termuhlen et al., 2011). Some sequelae, such as those that occur after radiotherapy, are considered to be complications or side effects that occur within a considerably long latent period following the completion of treatment. These complications may be more dangerous than the side effects associated with other treatment modalities because they occur in growing children. Therefore, it is necessary to accurately classify the tumor characteristics at the molecular level, which can avoid inappropriate use of aggressive treatment (e.g., chemotherapy and radiotherapy) among low-risk patients, and then reduce the development of treatment-related chronic disease.

Pediatric embryonal neoplasms, such as WT, typically present a limited number of genetic aberrations (Gadd et al., 2017). DNA methylation, which is one of the most intensively studied epigenetic modifications, describes the addition of a methyl group to the cytosine bases of a DNA sequence, usually to CpG dinucleotides. Some regions of DNA with a high G + C content (greater than 50%) and observed CpG/expected CpG ratio of greater or equal to 0.6, if > 200 bp, are defined as “CpG Islands” and are often found in the promoter regions of active genes (Gardiner-Garden and Frommer, 1987; Voisin et al., 2015). To date, DNA methylation has been demonstrated to be involved in the pathogenesis of many diseases, including tumor development. Previous studies have shown that genome-wide dysregulation of DNA methylation is associated with WT patients who display high-risk histology (Brzezinski et al., 2021). The early and prevalent events in WT are associated with DNA methylation changes affected many common cellular functions, most of which are involved in the epigenetic regulation of early renal development or transcription. Therefore, it is important to evaluate the prognostic potential of this molecular feature for classification determination, prognosis assessment and identification of appropriate treatment strategies.

In this study, we systematically characterized the DNA methylation levels in the Therapeutically Applicable Research to Generate Effective Treatments (TARGET) database to identify biological and clinical subgroups of WT in children. Our datasets and the classification regimens were then used to develop a prognostic model that integrates several representative DNA methylation markers to classify children with WT into high- and low-risk groups. Comprehensive profiling that includes stage, histology and DNA methylation analysis during clinical diagnosis may help clinicians to evaluate the disease progression and select appropriate therapeutic strategies to treat WT in children. Numerous studies have focused on the pivotal role played by the tumor microenvironment in the initiation and progression of WT (Maturu et al., 2017; Fiore et al., 2021; Stahl et al., 2021), therefore, we also analyzed the level of immune infiltration in high- and low-risk groups to evaluate the potential correlation between methylation and immune infiltration in WT.

## Materials and Methods

### Data Collection

The methylation data (Illumina Infinium HumanMethylation450 BeadChip), RNA-sequencing (RNA-seq) transcriptome data, and corresponding clinical information for WT samples were downloaded from the TARGET database (https://ocg.cancer.gov/programs/target). The data were downloaded on July 24, 2020. The clinical information contains patient de-identified information, including sex, age, event, tumor stage, and histological type, with event referring to the endpoint of WT patients, including none, relapse, and progression. Samples with unknown clinical information were removed, and the remaining information for a total of 124 children with WT was included in our analysis. The data acquired from the TARGET database was preprocessed, including removing probe sets that were absent in more than 70% of the WT samples. The cross-reactive CpG sites and polymorphic CpGs sites were discarded (Chen et al., 2013). In addition, the CpG sites from the sex chromosomes and single-nucleotide polymorphisms (SNPs; a list of SNPs that could potentially affect the methylation array results if present in the test population, can be found at https://support.illumina.com/downloads/infinium_hd_methylation_snp_list.html) were also discarded. In the sva R package, the k-nearest neighbor (KNN) imputation approach was used to estimate the other unidentified probes (Zhang et al., 2018). All methylation data were normalized by the “limma” package in R before further analysis (Ritchie et al., 2015).

### Identification and Molecular Subtyping of CpG Sites With Important Prognostic Significance in WT

The univariate Cox regression model in R package, “survival,” was applied for the selection of CpG sites with prognostic value, using *p* < 0.001 as the threshold for significance. To determine the CpG sites with independent prognostic value, the significant prognostic factors identified in the univariate analysis were analyzed by using forward stepwise analysis in a multivariate Cox proportional hazards model, in which the clinical parameters (sex, age, event, tumor stage, and histological type) were regarded as covariates. *p* < 0.001 was set as the significance threshold. Finally, significant independent prognosis-related CpG sites identified in both the univariate and multivariate analyses were selected as characteristic biomarkers for further analysis. To identify different DNA methylation prognostic molecule subtypes in WT, the k-means algorithm was used to perform unsupervised hierarchical clustering of the CpG sites, which were identified as independent prognostic factors in the multivariate analysis. The k-means clustering algorithm randomly selects K objects as the initial clustering center. Euclidean distances are used to calculate similar distances between samples, and k-means are used to perform clustering. The cumulative distribution function (CDF) was used to judge the optimal cluster number. The k-means algorithm was realized by the “kmeans” function in the ConcensusClusterPlus R packet.

### Clinical Characteristics and Molecular Assay Data for Molecular Subtypes

Based on previous clustering results, the clinical characteristics and differential analysis of methylation levels were analyzed for each subgroup. The overall survival (OS) curve of the WT subgroups defined by the DNA methylation modification levels was constructed using the Kaplan-Meier method, and a logarithmic rank test was used to determine significant differences among clusters. Histograms were drawn with the ggplot package to reflect the characteristics of DNA methylation subgroups according to age, sex, tumor stage, event, and histological type.

### Genome Annotations and Pathway Analysis of Prognosis-Associated Sites

Genome annotations of all independent prognosis-associated CpG sites we identified were performed to obtain the genes in which these CpG sites are located. The genome annotation files were obtained from the methylation data in the TARGET database. To determine the relevance and correlations between DNA methylation levels and gene expression levels, we extracted gene expression profiles from the downloaded data and generated an expression profile heat map. Finally, Gene Ontology (GO) enrichment analysis and the Kyoto Encyclopedia of Genes and Genomes (KEGG) functional enrichment analysis were performed on the genes corresponding to these CpG sites using clusterprofile package in R software. GO and KEGG pathways with *p*-values less than 0.05 were considered significant.

### Screening of Specific CpG Sites Between Molecular Subtypes

To study the differences between WT classifications, based on methylation modification levels, we analyzed the differences in 312 CpG sites across different subtypes. False discovery rate (FDR) and fold change (FC) values for the corresponding CpG sites in each subtype were calculated, in turn. FDR <0.05 and |log_2_FC| > 1 were set as the thresholds for identifying unique CpG sites in each cluster. Subsequently, we screened out the CpG sites that showed differences across different subtypes for subsequent model construction. In addition, differences in the frequencies of each methylation site in each subtype were examined. The site was obtained showing only one subtype difference, which was conducted as the specific CpG sites voiced in this subtype. To further express differences in the CpG sites between the different subtypes, heatmaps of differentially expressed CpG sites were generated using the “ComplexHeatmap” package (Gu et al., 2016).

### Construction of the Prognosis Prediction Model

Using the identified specifically CpG sites, a prognostic prediction model was constructed based on the multivariate Cox regression analysis. The expression of each gene and its coefficient, which was determined using the Cox hazard ratio (HR), were multiplied to calculate a risk score for each patient. The WT cohort was categorized into high- or low-risk groups divided by the median risk score. Survival analysis, performed in the “survival” R package, showed the OS of patients with different risk scores, including those with different clinical characteristics. The sensitivity and specificity of the model were evaluated using receiver operating characteristic (ROC) curves and area under the curve (AUC) analyses. Univariate and multivariate Cox regression models were applied to investigate independent prognostic factors.

### DNA Methylation Data Were Used to Estimate Immune Cell Infiltration

To evaluate the potential correlation between methylation and immune infiltration in WT, the infiltration levels of each tumor-infiltrating immune cell (TIIC) type were calculated by the *in-silico* deconvolution of DNA methylation data in the “EpiDISH” R package (Teschendorff et al., 2017). The immunological cell levels of 7 cell subtypes (B cells, CD4^+^ T cells, CD8^+^ T cells, natural killer [NK] cells, monocytes, neutrophils, and eosinophils) were evaluated based on the 450 k DNAm array. The histogram and violin plot display the composition of invading immune cells for every sample, allowing for the effective comparison of the relative proportions of immune cells between the high-risk and low-risk groups. The Wilcoxon test was applied to compare the immune cell scores between the two groups. A *p*-value less than 0.05 was considered significant.

## Results

### Identification of Characteristic CpG Sites According to the Prognosis of WT Patients

The univariate Cox proportional hazards regression model was performed using the R package “survival” to analyze each methylation site and survival status, with the significance threshold established at *p* < 0.001. We obtained 2,092 sites with significant prognostic effects, the top 20 of which are shown in Table 1. Sex, age, event, tumor stage, and histological type were also applied to the univariate Cox analysis, which revealed the following: sex (*p* = 0.04858), age (*p* = 0.7567), event (*p* = 0.004160), tumor stage (*p* = 0.004708), and histological type (*p* = 0.2603). The 2,092 CpG sites identified in the proportional hazards model were introduced into the multivariate Cox proportional risk regression model to select independent prognostic biomarkers, which included sex, age, event, tumor stage, and histological type as covariates. As a result, 312 significant CpG sites independent of other clinical information were obtained for further prognostic subgroup analysis (Supplementary Table S1).

**TABLE 1:** The top 20 among 2,092 independent prognostic methylation sites.

ID	HR	95% confidence down limit	95% confidence upper limit	*p* Value
cg23356505	0.000161815	5.91E-06	0.004433355	2.37E-07
cg06046705	4.13E+31	2.63572E+19	6.48E+43	3.75E-07
cg18376163	77628309593	4712054.035	1.27888E+15	4.16E-07
cg00150882	9.11207E+11	21300801.06	3.89797E+16	4.16E-07
cg01607897	5.00E-60	4.87E-83	5.13E-37	4.40E-07
cg25456549	6.87E+20	4.67282E+12	1.01E+29	5.72E-07
cg03140678	4.06E-50	1.76E-69	9.40E-31	5.76E-07
cg14101501	2.91E+46	1.12E+28	7.55E+64	7.60E-07
cg26036806	1.31E+32	2.02732E+19	8.46E+44	8.93E-07
cg17251433	327.5474238	32.42261519	3309.027178	9.20E-07
cg08435936	2.62E+52	2.83E+31	2.42E+73	9.57E-07
cg11059651	9.14E+37	5.99E+22	1.40E+53	9.58E-07
cg12693634	5.18E+44	6.14E+26	4.37E+62	1.01E-06
cg04940109	1.78884E+14	318289556.7	1.01E+20	1.18E-06
cg00829406	1.35E+24	1.84622E+14	9.83E+33	1.63E-06
cg07904475	357,527.8872	1844.815012	69289435.17	1.95E-06
cg13945578	1.48635E+12	14182073.28	1.55776E+17	2.01E-06
cg10425506	2.54E+25	8.31148E+14	7.77E+35	2.05E-06
cg10945313	1.16366E+14	147945445.7	9.1528E+19	2.93E-06
cg08521800	7.59E+21	5.07045E+12	1.14E+31	2.95E-06

HR:hazard ratio.

### Identification of Four Clinical Subtypes Among WT Patients According to Consensus Cluster Analysis

**FIGURE 1:**
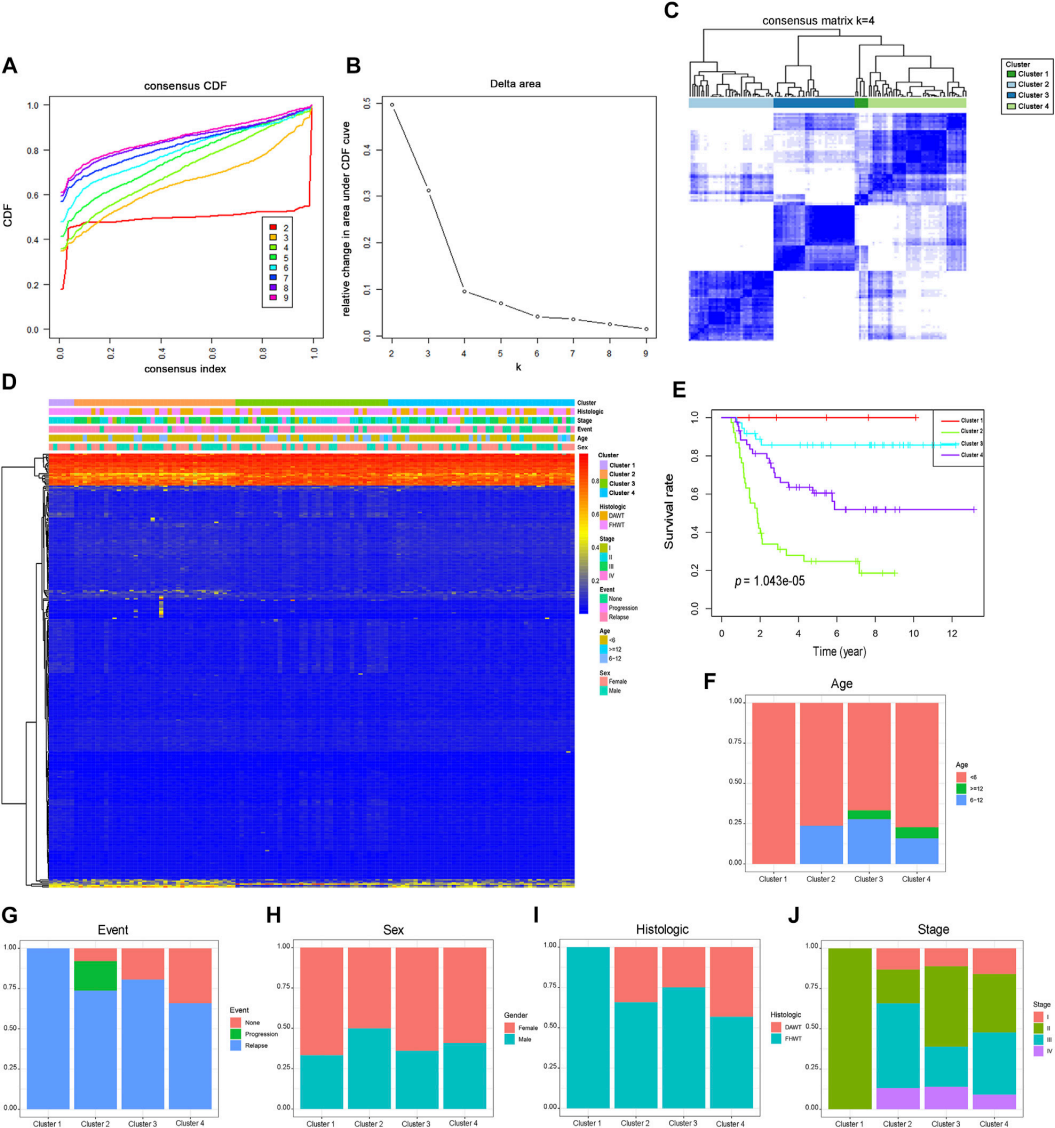
Consensus clustering of different DNA methylation prognostic subgroups, survival analysis and clinical features in Wilms tumor. **(A)** Consensus clustering cumulative distribution function (CDF), with k = 2 to 9. **(B)** Relative changes in the CDF area curve for k = 2 to 9. **(C)** Clustering heatmap for k = 4. **(D)** The methylation heatmap for 312 sites in four clusters. **(E)**. Kaplan-Meier survival analysis of the four clusters. Different proportions of ages **(F)**, event **(G)**, sex **(H)**, histological types **(I)**, and stage scores **(J)** in the four clusters. FHWT: favorable histology Wilms tumor; DAWT: diffuse anaplastic Wilms tumor.

All samples in the cohort were classified by the unsupervised hierarchical clustering of the 312 prognosis-related sites (Figure 1A). Further surveillance of the CDF delta area curve revealed that the clustering result was stable when the selection was 4, as the changing trends in the CDF AUC began to moderate when more than four classes were used (Figure 1B). Based on the similar methylation levels, k = 4 was demonstrated to be the most appropriate choice, dividing the WT patient cohort into four clusters. The result of stable clustering was divided into four clusters to establish a consensus matrix graph based on the results of consistent clustering. A color-coded heatmap was constructed to demonstrate a clearly defined 4-block configuration, displayed as blue blocks arranged along the diagonal line on a white background (Figure 1C). The consensus matrix shows that when K is 4, the differences within the clinical typing groups are small, but the differences between groups are large. Furthermore, the heatmaps were generated using DNA methylation data and classification according to sex, age, event, tumor stage, and histological type (Figure 1D). Sites located in different categories displayed different methylation patterns. CpGs beta values in the range from 0 to 0.2 were defined as low methylation and those from 0.8 to 1 as high methylation. The heatmap analysis revealed that most of the CpG sites were low methylation levels. We further examined the prognostic status of distinct WT subtypes. Kaplan-Meier analyses demonstrated that Cluster one had the best prognosis, whereas Cluster two was associated with the worst prognosis (Figure 1E), suggesting that a favorable prognosis may be associated with higher levels of DNA methylation. The age, event, sex, histological type, and tumor stage distributions were analyzed for the four molecular subtypes (Figures 1F–J). Different methylation types revealed differences in age, event, sex, tumor stage, and histological type, indicating that DNA methylation can be used as a biomarker for the clinical classification of WT in children. The Cluster one subtype was associated with younger age and were all classified as FHWT, which may explain the good prognosis associated with this group. These findings indicated that the methylation spectrum could be used to explain the etiology of WT and contribute to the development of the best clinical treatment plan.

### Gene Expression and Pathway Enrichment Analysis of CpG Sites With Prognostic Implications

**FIGURE 2:**
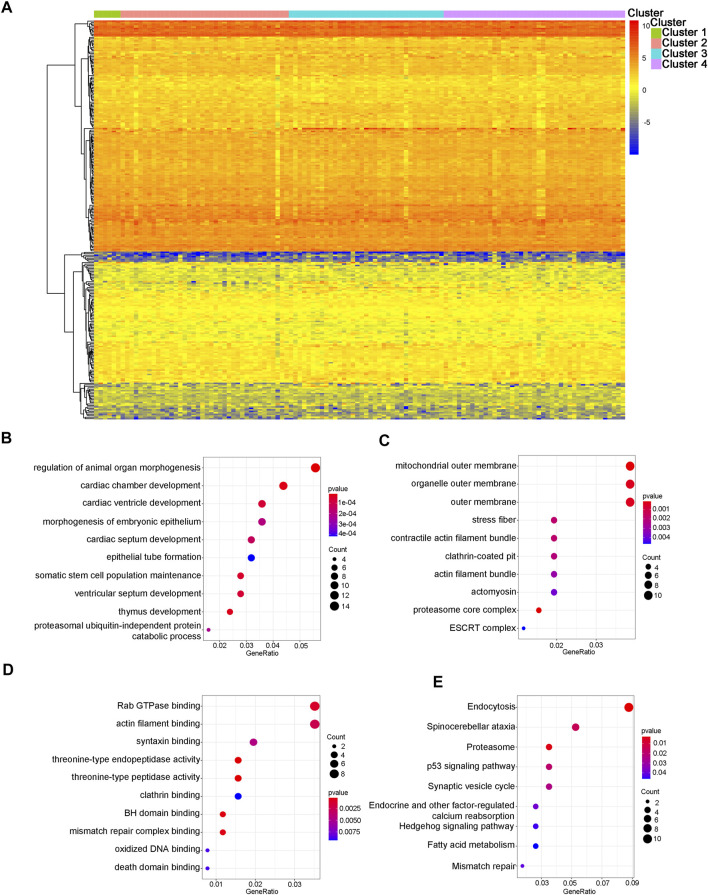
Gene annotations and functional enrichment analysis. **(A)** The heatmap of methylation modification levels for 305 corresponding genes associated with different CpG sites. The results of enrichment analyses for these 305 genes. **(B)** The top 10 significant GO-BP results. **(C)** The top 10 significant GO-CC results. **(D)** The top 10 significant GO-MF results. **(E)** The nine significant signaling pathways from the KEGG analysis. GO: gene ontology; BP: biological processes; CC: cellular components; MF: molecular functions; KEGG: Kyoto Encyclopedia of Genes and Genomes.

We further analyzed the differences in 312 CpG sites across each WT subtype and mapped the 305 genes associated with these CpG sites. 52.56% of the methylation sites were derived from the gene body, while 47.44% of the methylation sites were derived from regulatory regions of thees gene. The expression data of genes associated with specific CpG sites were extracted from the TARGET database and used to plot a heat map of the gene expression spectrum (Figure 2A). Because biologically relevant processes may be affected by epigenetic regulation at these candidate CpG sites, we examined the potential functional significance of genes associated with the independent prognostic CpG sites. The biological functions and pathways were explored using GO gene sets and KEGG datebase as background, respectively. Figures 2B–E shows that the genes associated with specific CpG sites were primarily concentrated in biological pathways that were strongly related to tumors, such as the p53 signaling pathway and Rab GTPase binding.

### Identification of Specific CpG Sites Between Subtypes

**FIGURE 3:**
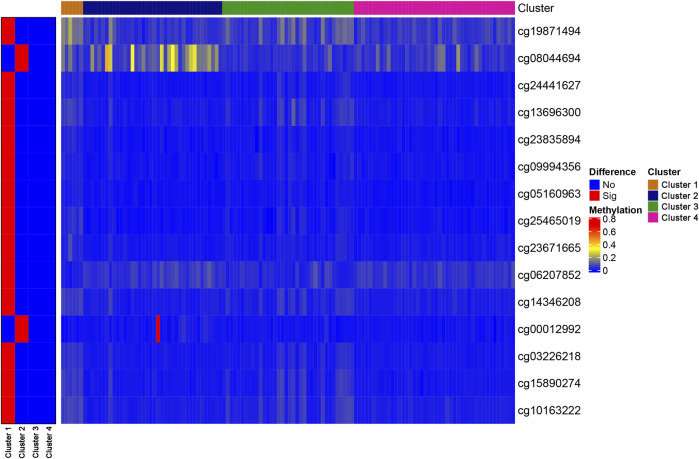
The specific CpG sites associated with different DNA methylation prognostic subtypes. Heat map for the modification levels of these CpG sites is shown on the right. The red and blue represent hyper/hypomethylated CpG sites, respectively.

To identify unique CpG sites that were specific to each subtype, we set the FDR to 0.05 and the logFC to 1, which resulted in the identification of 15 subtype-specific CpG sites. All of the specific CpG sites associated with each DNA methylation cluster are displayed on the left-hand side of Figure 3, which shows that Cluster one contained the most subtype-specific CpG sites, most of which were hypomethylated. However, few specific CpG sites displayed hypermethylation, and no specific CpG sites were detected for Cluster 4. The right-hand side of Figure 3 shows the degree of methylation observed at all specific CpG sites. Other clusters were associated with a few specific CpG sites, most of which were hypermethylated.

### Establishment of Prognostic Prediction Models

First, to determine the predictive abilities of specific CpG sites, the specific CpG sites in Cluster one associated with the best prognostic outcomes were assessed. These CpG sites were constructed a prognostic signature. However, because of the large number of these CpG sites, this signature was not deemed to be ideal for clinical detection. Therefore, a multivariate Cox regression analysis was performed by applying the “survival” package in R software to construct and optimize the model and to further reduce the range of evaluated CpG sites while maintaining a high degree of accuracy. Stepwise model selection was further performed, using the Akaike Information Criterion (AIC) to avoid over-fitting, to select the final list of sites. Then, in the analysis of the multivariate Cox proportional hazards model, the value of the weighted coefficient was determined by the regression coefficient for the corresponding sites. The risk score model was evaluated using a formula based on the results of the multivariate regression analysis, as follows:

The risk score formula for this model was (−78.14 × expression value of cg23671665) + (19.30 × expression value of cg06207852) + (−49.39 × expression value of cg14346208).

**FIGURE 4:**
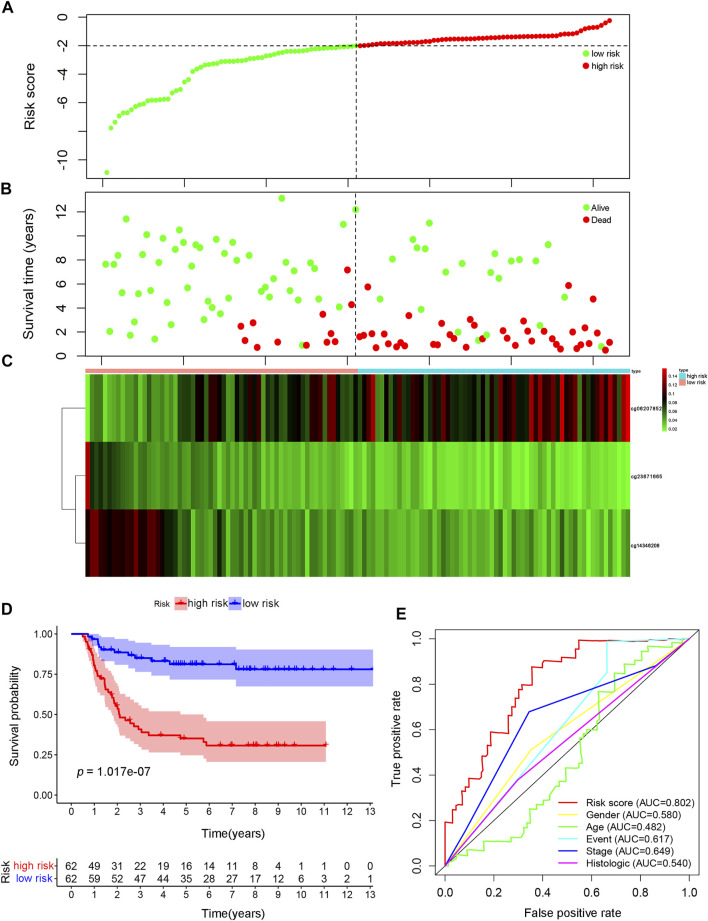
Construction of the prognosis prediction model using specific CpG sites in Wilms tumor. **(A)** The distributions of risk scores. **(B)** Correlation between risk score and overall survival. **(C)** Significant differences were found for the methylation pattern between the high- and low-risk groups. **(D)** The survival difference was based on Kaplan-Meier analysis between high-risk-score and low-risk-score groups. **(E)** The result of ROC analysis for the Wilms tumor cohort, indicating the prediction efficiency of the prognostic signature. ROC: receiver operating characteristic.

The methylation modification levels for these CpG sites were obtained from the TARGET queue and substituted into the model for calculation. Patients were ranked on the basis of the calculated risk score. From the performance shown in Figure 4A, the risk score was primarily distributed in the range of −10 to 0, with increasing scores indicating greater risk. Increasing risk scores were associated with significant decreases in the OS of the patients due to WT (Figure 4B). Increasing risk scores were associated with the decreased methylation of cg23671665, a gradual decrease in the methylation of cg14346208 decreased, and a gradual increase in the methylation of cg06207852, in addition to a decrease in OS (Figure 4C). The samples were divided into a high-risk group (risk score > median score) and a low-risk group (risk score < median score), with the median risk score used as the grouping threshold, to explored differences in prognosis between the two groups. The prognosis of the high-risk group was significantly worse than that of the low-risk group, as shown in Figure 4D (*p* = 1.017e−07). The AUC value of the prognostic signature model was 0.802, whereas the AUC values of all other models were <0.7, indicating that this model has the best prognostic prediction efficiency (Figure 4E).

### The Independent Prognostic Value of the Risk Score

**FIGURE 5:**
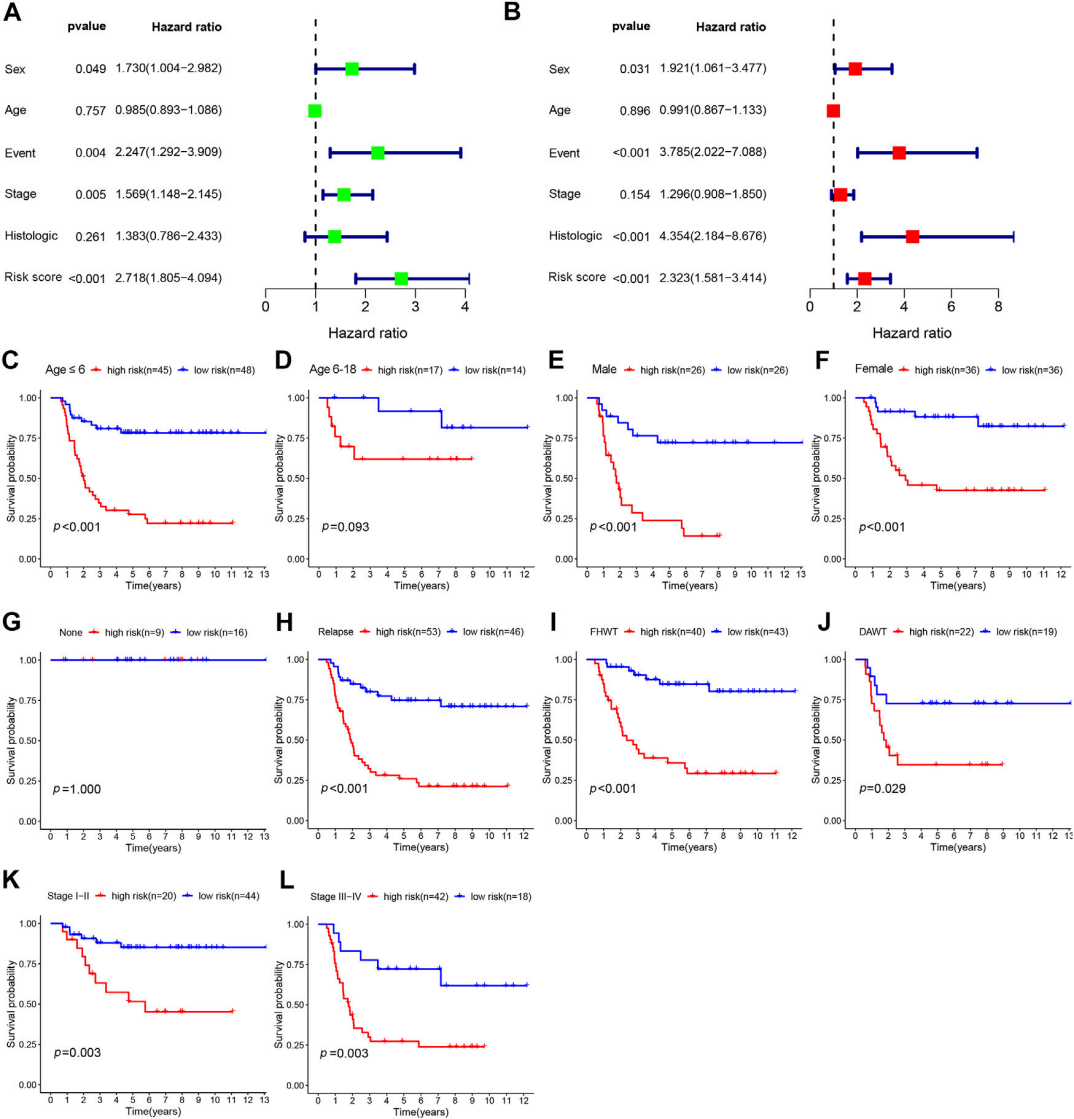
The prognostic analysis of risk score and different clinical features. **(A)** Univariate analysis was used to identify factors associated with OS. **(B)** Multivariate analysis was performed to reveal independent prognostic factors. Kaplan–Meier survival analysis for high- and low-risk groups according to different clinical features, including age **(C, D)**, sex **(E, F)**, event **(G, H)**, histologic type **(I, J)**, and stage **(K, L)**. OS: overall survival; FHWT: favorable histology Wilms tumor; DAWT: diffuse anaplastic Wilms tumor.

To determine whether the risk score could serve as an independent prognostic predictor for WT, univariate and multivariate Cox regression analyses were performed. The univariate analysis revealed that sex (*p* = 0.049, HR: 1.730, 95% confidence interval [CI]: 1.004–2.982), event (*p* = 0.004, HR: 2.247, 95% CI: 1.292–3.909), stage (*p* = 0.005, HR: 1.569, 95% CI: 1.1148–2.145), and risk score (*p* < 0.001, HR: 2.718, 95% CI: 1.805–4.094) were significantly associated with OS (Figure 5A). After correction for other confounding factors, sex (*p* = 0.031, HR: 1.921, 95% CI: 1.061–3.477), event (*p* < 0.001, HR: 3.785, 95% CI: 2.022–7.088), histological type (*p* < 0.001, HR: 4.354, 95% CI: 2.184–8.676), and risk score (*p* < 0.001, HR; 2.323, 95% CI; 1.581–3.414) were independent predictive factors for OS in the multivariate Cox regression analysis (Figure 5B).

In the subgroup analyses, we further explored the prognostic differences between patients with high- and low-risk scores according to different clinical features, including age, sex, event, histological type, and stage. As shown in Figure 5C-L, the Kaplan–Meier survival analysis demonstrated that survival was remarkably reduced among high-risk patients compared with low-risk patients for male patients (*p* < 0.001), female patients (*p* < 0.001), those aged ≤6 years (*p* < 0.001), those with relapse (*p* < 0.001), diffuse anaplastic WT (DAWT; *p* = 0.029), FHWT (*p* < 0.001), WT stages I–II (*p* = 0.003), and WT stages III–IV (*p* = 0.003). However, no significant discrepancy in OS rate was observed between high- and low-risk patients among patients with no relapse or progression (*p* = 1) or among patients aged 6–18 years (*p* = 0.093), which may be associated with the small sample size. These results suggested that the prognosis signature can predict the prognosis of patients accurately across various clinical features.

### Association Between the Risk Score and the Composition of TIICs

**FIGURE 6:**
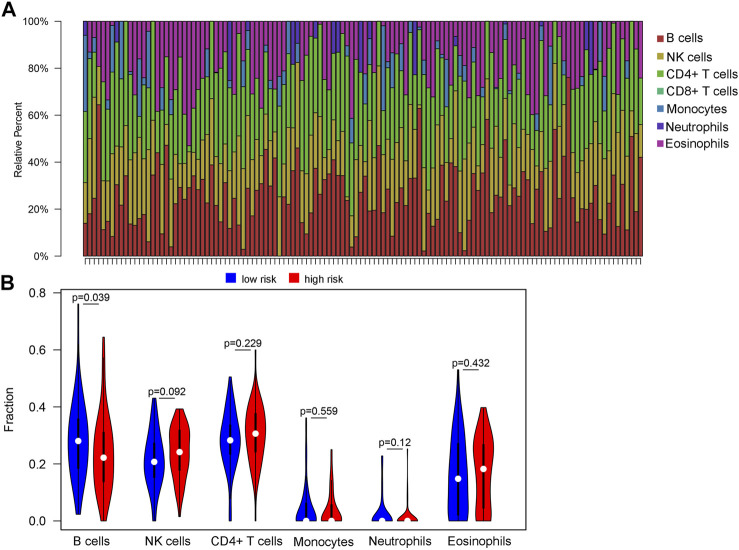
Variations in the immune cell infiltration between high-risk-score and low-risk-score groups. **(A)** Histogram of estimated immune cell compositions obtained using EpiDISH. **(B)** The violin plot output, showing the compositions and differences in invading immune cells between the high risk score and low risk score groups.

We attempted to investigate the relationship between the risk score and diverse immune infiltrating cells in the tumor microenvironment of WT. By using two distinct DNAm reference matrices to examine the discrepancies in the ratios of seven types of immune cells between the high-risk group and the low-risk group, the specific cell types driving differential methylation were identified. The outcomes of the seven immune cell subsets are revealed in Figure 6A, which shows that B cells and CD4^+^ T cells were relatively abundant in cancer tissues, whereas CD8^+^ T cells were not expressed in cancer tissues. The only immune cells that were significantly different between groups divided by risk score were B cells. Specifically, B cells (*p* = 0.039) were present at higher proportions in the low-risk group than in the high-risk group (Figure 6B).

## Discussion

The incidence of WT, the most commonly identified tumor that arises from the kidney in children, has steadily increased over time, with a disproportionate increase observed among male children (Nakata et al., 2020). Increasing evidence suggests that a greater than 90% 5-years survival rate can be achieved among children diagnosed with WT after receiving active anticancer treatments (Szychot et al., 2014), such as upfront nephrectomy, as described by the Children’s Oncology Group (COG), and nephrectomy following chemotherapy, as described by the studies reported by the International Society of Pediatric Oncology (SIOP). However, many therapeutic challenges remain to be resolved. There are still some WT patients whose survival rate is less than 90% (Dome et al., 2015). WT survivors continue to have a higher risk of developing chronic health and late mortality beyond that anticipated for the general population (Suh et al., 2020). Child survivors of WT are more likely to require special education or present with mental health concerns (Foster et al., 2021). Childhood cancer survivors are at increased risk of adverse adjuvant therapy effects, as the administration of systematic chemotherapy may influence the development of cardiovascular conditions (Mulrooney et al., 2009). Consequently, clinically viable biomarkers are necessary to better distinguish between high and low risk WT patients, which can aid clinicians in the clinical strategies and accurate treatment of WT. This is very valuable for avoiding overtreatment and reducing related complications.

Currently, increasing evidence suggests that a series of gene methylations may be associated with the development and progression of WT. Moreover, patients who present with the genome-wide dysregulation of DNA methylation have been associated with high-risk histology tumors and the occurrence of relapse (Foster et al., 2021), indicating that abnormal DNA methylation, either alone or in combination with the existing surveillance methods, may serve as a potential epigenetic biomarker or therapeutic target for WT patients. Currently, CpG sites that have been shown to be associated with potential WT pathogenesis include Wilms tumor 1 (*WT1*) (Brzezinski et al., 2021), *WTX* (Pelletier et al., 1991), *TP53* (Rivera et al., 2007), and *11p15* (Andrade et al., 2014), and the methylation of some of these genes can be detected in peripheral blood (Fiala et al., 2020). In recent studies, methylation patterns have been studied and recommended as a potential method for disease classification and prognostic assessment. However, in big data samples from patients with WT, whether these methylation signatures have clinical significance in tumor classification, survival, and prognosis has remained uncertain.

Consequently, we conducted this study to determine the detailed epigenomic classification of WT according to the methylation pattern. CpG sites associated with prognosis were obtained and classified by sex, age, event, tumor stage, and histological type. Expression data for 312 independent prognosis-associated CpG sites (*p* < 0.05) corresponding to WT patients were obtained from the TARGET database and subjected to unsupervised clustering analysis. We obtained a detailed classification of the WT epigenomes by applying cluster analysis. WT can be divided into four distinct molecular subgroups, among which Cluster one showed the best prognosis, which may be associated with the characteristics of younger age, a lower stage, and better tissue type by comparison of clinical information in different subgroups. As a consequence, the classification scheme can be used for the molecular stratification of individual tumors, which may allow clinicians to reassess patient treatment strategies and clarify the biological mechanisms underlying the occurrence and development of WT. The integration of prognosis-related CpG sites can generate more accurate results and facilitate preferable risk stratification. A scoring system based on methylation can be applied to clinical practice to stratify risk in patients with early WT. A risk score was established to predict the prognosis of WT patients and guide decisions regarding the application of adjuvant therapy. We identified three CpG sites that were specific to Cluster one in WT and established a prognostic risk score-based model according to the methylation status of these three sites. The results of AUC analysis among the WT cohort indicated the correctness and reliability of the prediction model. The risk score remained an effective independent predictive factor even when the model was corrected for other factors (*p* < 0.05).

Increasing studies have shown that TIICs in WT are related to the initiation and prognosis of WT (Maturu et al., 2017; Fiore et al., 2021; Stahl et al., 2021). Oleinika et al. found that immune dysregulation contributes to the pathogenesis of many kidney diseases, regardless of antibody involvement (Oleinika et al., 2019). Therefore, the exploration of immune infiltration in WT remains necessary and may affect the outcomes of immunotherapy. Although the mechanism of B cells in WT remains unclear, our study suggested a significant correlation between decreased B lymphocyte infiltration and increased risk score. A pronounced decrease in the B lymphocyte numbers was observed in the high-risk score group (*p* = 0.039). B lymphocytes perform a variety of immunological functions, which play important roles in the production and secretion of antibodies during humoral immunity (Franchina et al., 2018). Some studies have suggested that B lymphocytes may exert an anti-tumor effect, whereas others have shown that B lymphocytes are tumor-promoting (Tsou et al., 2016; Largeot et al., 2019). In one study of metastatic melanoma and renal cell carcinoma, tumor-infiltrating B lymphocytes played an active role in anti-tumor immunity (Helmink et al., 2020). Similar studies have found that the presence of peritumoral B lymphocytes correlates with healthy long-term survivorship following WT (Eckschlager et al., 2009). Importantly, due to their involvement in the release of cytokines and other mechanisms, B lymphocytes are likely capable of interacting with the components of both immune cells and non-immune cells in the tissue microenvironment (Largeot et al., 2019). The observation of antibodies directed against tumor antigens in many cancer patients suggests that B lymphocytes may potentially produce antibodies that promote tumor clearance (Reuschenbach et al., 2009). B lymphocytes also produce several cytokines and chemokines, which recruit other immune cells. Memory B lymphocytes may act as antigen-presenting cells (APCs), inducting tumor-specific T cells to promote anti-tumor immunity. Therefore, future studies should examine the feasibility of using the tumor regulatory potential of B lymphocytes to improve WT immunotherapy.

Inevitably, this study is associated with some innate limitations that must be addressed. First, this study was performed as a retrospective study based on the results of a single-institutional publicly available online database, sample size limitation with no other database cohorts available for validation. Moreover, most of the WT patients in the target database were white. The limited racial diversity represented by the data may bias the results toward outcomes that are specific to the white population. Additionally, it is necessary to further investigate the regulatory mechanisms to confirm the functions of these CpG sites and their effects on tumor immune microenvironment.

## Conclusion

We classified WT into four prognostic subgroups based on the TARGET methylation spectrum, which efficiently identified high-risk WT patients. A prognostic methylation risk score was developed that was significantly associated with the unfavorable clinical outcomes, indicating that this prognostic signature might be used to guide clinicians in devising reasonable treatment plans and evaluating their efficacy. However, further independent studies and large scale prospective studies are needed to validate and advance this hypothesis.

## Data Availability

The results published here are in whole or part based upon data generated by the Therapeutically Applicable Research to Generate Effective Treatments (https://ocg.cancer.gov/programs/target) initiative, phs000471. The data used for this analysis are available at https://portal.gdc.cancer.gov/projects/TARGET-WT.

## Abbreviations

AUC, area under the curve; AIC, Akaike Information Criterion; COG, Children’s Oncology Group, CDF; cumulative distribution function; DAWT, diffuse anaplastic Wilms tumor; DEGs, differentially expressed genes, FDR, false discovery rate; FHWT, favorable histology Wilms tumor; FC, fold change; GO, Gene Ontology; HR, hazard ratio; KEGG, Kyoto Encyclopedia of Genes and Genomes; NK cell, natural killing cell; OS, overall survival; ROC, receiver operating characteristic; SIOP, Society of Pediatric Oncology; TARGET, Therapeutically Applicable Research to Generate Effective Treatment; TIICs, tumor-infiltrating immune cells; WT, Wilms tumor.
